# Gut Microbiota, the Ketogenic Diet and Epilepsy

**DOI:** 10.15844/pedneurbriefs-32-10

**Published:** 2018-09-21

**Authors:** Egidio Spinelli, Robyn Blackford

**Affiliations:** 1 Epilepsy Center and Division of Neurology, Ann & Robert H. Lurie Children’s Hospital of Chicago, Chicago, IL; 2 Department of Clinical Nutrition, Ann & Robert H. Lurie Children’s Hospital of Chicago, Chicago, IL

**Keywords:** Ketogenic Diet, Gut Microbiome, Epilepsy

## Abstract

Investigators from the Department of Pediatric Neurology at the Children’s Hospital of Fudan University assessed the gut microbiome in pediatric patients with intractable non-lesional epilepsy who were treated with the ketogenic diet (KD) comparing differences between responders and non-responders over a period of 6 months.

Investigators from the Department of Pediatric Neurology at the Children’s Hospital of Fudan University assessed the gut microbiome in pediatric patients with intractable non-lesional epilepsy who were treated with the ketogenic diet (KD) comparing differences between responders and non-responders over a period of 6 months. Twenty (14 males, 6 females) patients (median age at enrollment: 4.2 years, range 1.2-10.3 years) were treated with a classic 4:1 ratio ketogenic diet. All patients were treated with at least 2 antiepileptic medications and had a normal MRI brain with no family history of seizures. Diagnoses included: Dravet Syndrome (5 patients), Lennox Gastaut Syndrome (3 patients), West Syndrome (3 patients) and unclassified (9 patients). After 6 months of KD treatment, 10 patients were considered responders (decreased in seizure frequency of ≥ 50%) with concomitant improvement in electroencephalography. Gut microbiome analysis was performed by 16s rDNA sequencing on fecal samples before and after initiation of KD and species diversity was compared between responders before and after treatment. The researchers showed that there was an overall decrease in the mean species diversity after treatment and importantly, a difference between the variation of species between responders and non-responders. Further analysis of species composition before and after treatment showed a significant increase in *Bacteroides* and a decrease in *Firmicutes* and *Actinobacteria*. When comparing responders and non-responders, *Clostridiales*, *Clostridia*, *Ruminococcaeceae*, *Lahnospiraceaea*, *Alistipes* and *Tikenellacase* were significantly increased in non-responders.

The authors conclude the KD can reduce the diversity of intestinal bacteria and there are further differences in the composition between those that respond to the KD and those that do not. Furthermore, they suggest that intestinal bacteria may be used as a biomarker of efficacy as well as a potential therapeutic target in patients with refractory epilepsy. [1]

COMMENTARY. Approximately 9-24% of pediatric patients with epilepsy are refractory to more than 2 antiepileptic medications [2] and the KD can provide an effective adjunctive treatment helping to reduce the seizure burden [3]. To date, how the KD modulates epileptogenesis has been largely unknown, although a recent paper utilizing 2 mouse models of refractory epilepsy showed that those given antibiotics or reared in a germ-free environment were resistant to seizure protection from KD and that enrichment of the intestinal flora with bacteria that increase with KD treatment or transplantation with gut microbiota from KD treated mice helped with seizure control [4]. The timely work by Zhang et al. [1] suggests that there are differences in intestinal bacterial composition between pediatric patients with refractory epilepsy that do and not respond to the ketogenic diet. This study raises several questions: 1) How does gut bacteria affect epileptogenesis? 2) Can monitoring gut bacterial composition be used as a marker for treatment efficacy and, as is seen in mouse models, 3) Can altering bacterial composition be used as a therapeutic strategy? A large multicenter research study is needed to further expand these results and to better elucidate whether a potential microbe based treatment is a reasonable option in pediatric refractory epilepsy.

## Disclosures

The author(s) have declared that no competing interests exist.
